# Effect of Ni Content on Microstructure and Characterization of Cu-Ni-Sn Alloys

**DOI:** 10.3390/ma11071108

**Published:** 2018-06-29

**Authors:** Sanming Du, Xiaochao Wang, Zhen Li, Zhenghai Yang, Jingbo Wang

**Affiliations:** 1 National United Engineering Laboratory for Advanced Bearing Tribology, Henan University of Science and Technology, Luoyang 471023, China; xcwang710@163.com (X.W.); yzh772029@163.com (Z.Y.); 2 State Key Laboratory of Solid Lubrication, Lanzhou Institute of Chemical Physics, Chinese Academy of Sciences, Lanzhou 730000, China; jbwang@licp.cas.cn

**Keywords:** Cu-Ni-Sn alloys, mechanical properties, precipitate, microstructure, morphology

## Abstract

Cu-xNi-5Sn (wt %) alloys with a different Ni content were prepared by a powder metallurgy method. The effect of Ni content on the hardness and yield strength of Cu-xNi-5Sn (wt %) alloys was investigated. The microstructure, composition, and morphology of Cu-xNi-5Sn (wt %) alloys were observed by X-ray diffraction (XRD), scanning electron microscopy (SEM) with energy dispersive spectroscopy (EDS), and cold field emission scanning electron microscope (FESEM), respectively. Results indicate that the hardness and yield strength firstly increase and then decrease with the increase of Ni content and reach up to a maximum when Ni content is 12.5 wt %. Furthermore, the formation of the sandwich structure and needle-like phase is found in the grain, the grain boundary and intragranular precipitates are rich in both the Ni and Sn phase. The formation of the inerratic and suitable lamellar precipitates of sandwich structure and needle-like phase can be responsible for the good mechanical properties of the Cu-12.5Ni-5Sn alloy after aging treatment. The sandwich structure and need-like phase that were observed by FESEM can contribute to clarify the morphology of Cu-Ni-Sn alloys.

## 1. Introduction

The Cu-Ni-Sn alloys attracted wide interest in electronic and mechanical industries because of high strength, excellent stress relaxation, corrosion resistance, good thermal and electrical conductivity, and so on [1,2,3]. So, the Cu-Ni-Sn alloys are expected as candidates for the nocuous Cu-Be alloys with the high strength in the application of electrical springs, connectors, bearings, [3,4,5], etc.

It is known that the Cu-Ni-Sn alloys are strengthened by age-hardening. Some authors believed that the age-hardening of Cu-Ni-Sn alloys came from the spinodal decomposition [6,7]. However, the papers also demonstrated that the age-hardening derived from the formation of coherent DO_22_ phase [8,9,10]. So, many researches focused on the investigations of aging characterization of Cu-Ni-Sn alloys. Zhao and Notis [2,11] investigated the microstructures and the transformation kinetics of Cu-15Ni-8Sn alloy and Cu-7.5Ni-5Sn alloy under different aging conditions, and established a time-temperature-transformation diagram, respectively, which laid the foundation for the selection of heat treatment schedule in the future. Peng et al. [12] reported that the stress-strain curve of Cu-15Ni-8Sn alloy showed obvious serrated flows under the condition of dynamic strain aging and the pre-deformed alloy with dynamic strain aging generated Sn-enriched precipitation at a lower aging temperature when compared to the natural aging alloy without pre-deformation. Zhang et al. [13] investigated the phenomenon of age-hardening of Cu-15Ni-8Sn alloy coating, which was fabricated by laser cladding. Hermann et al. [4] studied the relationship between the microstructure and mechanical properties of Cu-15Ni-8Sn alloy, and he results indicated that the improvement of strength was attributed to grain-size reduction and internal stress fields that are caused by the age-hardening process. Up to now, much attention has focused on the improvement of mechanical properties and the microstructural evolution of Cu-Ni-Sn alloys with the same composition under different aging condition [14,15,16,17,18]. However, the studies on the effect of different alloy element content on the microstructure and characterization of Cu-Ni-Sn alloys under the same aging condition have been rarely reported yet. Sahu et al. [19] reported the microstructural characterization of Cu-15Ni-Sn alloys with different Sn content by X-ray diffraction. Shankar and Sellamuthu [20,21] investigated the effect of Sn or Ni content on hardness, aging temperature, and aging time of Cu-Ni-Sn alloys that were cast in metal mold. Therefore, the aim of the present work is to investigate the effect of Ni content on hardness, yield strength, microstructure and morphology of Cu-xNi-5Sn alloys under the same condition of aging treatment. The Cu-xNi-5Sn (wt %) alloys were prepared by the powder metallurgy technique in this paper to avoid Sn segregation. Morphology, composition, and microstructure of the alloys with the increasing of Ni content were examined to discuss the relationship between the microstructure and mechanical properties of alloys.

## 2. Materials and Methods 

In this investigation, the Cu-xNi-5Sn (wt %) alloys with different Ni content were prepared by powder metallurgy technique (denoted as C7.5NS, C10NS, C15NS, and C17.5NS, respectively). Commercially available Cu powder, Ni powder, and Sn powder were used as the starting materials. The nominally chemical compositions of each alloy were listed in Table 1. The raw metal powders were weighed according to the pre-designed weight ratios in the Table 1, and then the metal powders weighed were mechanically mixed using a three-dimensional vibration blender for 4–5 h. The mixed metal powders were firstly cool pressed under a pressure of 338 MPa, which was provided by four column hydraulic press (YB32-63C, Tianjin Tianduan Hydraulic Press Co., Tianjin, China) and then sintered at 885 °C for about 65 min in the tube furnace under hydrogen atmosphere; subsequently, aging treatment was performed at 400 °C for about 4 h in the tube furnace under hydrogen atmosphere. The sintered specimens before and after aging treatment were machined to the disk samples; finally, the disk samples were ground and then polished into metallographic samples for the following analyses and tests, then metallographic samples were ultrasonically washed in ethanol, and dried in a hot air. The metallographic specimens were etched by a mixture solution of 0.8 g CuSO_4_·5H_2_O, 10 mL HCl and 10 mL H_2_O. The microstructures, morphologies, and compositions of alloys were investigated by X-ray diffraction analysis (Empyrean, Panalytical, Almelo, The Netherlands) with Cu Kα radiation, scanning electron microscopy (JSM-5600LV, JEOL, Tokyo, Japan) with energy dispersive spectroscopy (EDS), and cold field emission scanning electron microscope (JSM-6701F, JEOL, Tokyo, Japan), respectively. The density of alloys after aging treatment was determined using a full automatic true density analyzer (AccuPyc 1300, Micromeritics Corp., Atianta, GA, USA) and are listed in Table 1. The Brinell hardness of the alloys before and after aging treatment was measured by a HBS-62.5 Brinell hardness tester with a load of 62.5 Kgf and a dwell time of 30 s according to Chinese National Standards GB/T 231.4-2009/ISO 6506-4: 2005, at least three tests were carried out and the average value was provided. Compression tests were conducted using a CMT5202 materials testing machine (Shenzhen Sans Material Test Instrument Co., Shenzhen, China), with a strain rate of 1.6 × 10^−3^ s^−1^ based on Chinese National Standards GB/T 7314-2005. Compressive specimens with gauge dimensions of 5 mm in diameter and 10 mm in length were prepared from the sintered samples before and after aging treatment, then at least three repeated measurements were performed for every specimen, and the average value was given.

## 3. Results and Discussion

### 3.1. Mechanical Properties and X-ray Diffraction (XRD) Patterns of the Alloys

**Figure 1:**
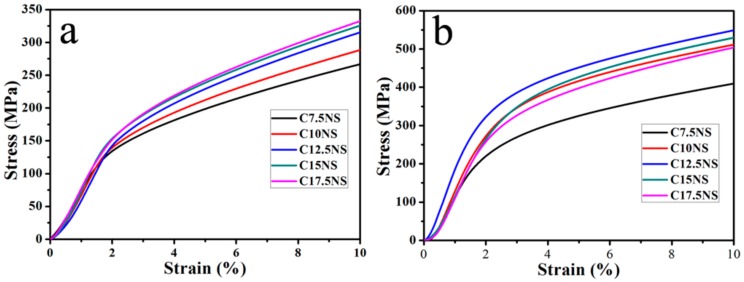
Compressive stress-strain curves of the alloys before (**a**) and after (**b**) aging treatment.

**Figure 2:**
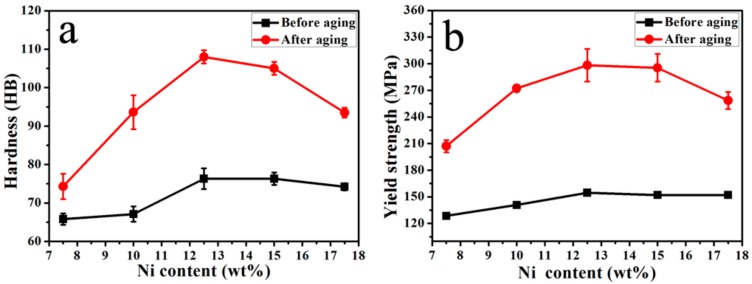
Effect of Ni content on mechanical properties of alloys before and after aging treatment: (**a**) hardness; (**b**) yield strength.

The densities of Cu-xNi-5Sn alloys with different Ni content are shown in Table 1. As shown in Table 1, the theoretical densities of the alloys are nearly identical whereas the measured densities of the alloys gradually increase with increasing Ni content. This indicates that the relative density of alloys increases with the increase of Ni content. Figure 1 shows that the compressive stress-strain curves of the alloys before and after aging treatment. It can be found lots of plastic deformation in the compression. It can indicate the good formability of alloys. It can be found that the yield strength after aging treatment increases compared with that before aging treatment. The effect of Ni content on hardness and yield strength of the alloys before and after aging treatment is shown in Figure 2. It can be seen from Figure 2a that the hardness of alloys before and after aging treatment firstly increases and then decreases with the increase of Ni content, and reach up to the maximum value of 76 and 108 HB when Ni content of the alloy is 12.5 wt %, respectively. For the same component alloy, the hardness of alloy after aging treatment is remarkably higher than that of alloy before aging treatment. It can be seen from Figure 2b that the yield strength of alloys before and after aging treatment firstly increases and then decreases with the increase of Ni content and reaches up to the maximum value of 154 and 298 MPa when Ni content of the alloy is 12.5 wt %, respectively. For the same component alloy, the yield strength after aging treatment was almost two times higher than that before aging treatment. It can be found that the hardness and yield strength of alloys before aging treatment change slightly with the increase of Ni content. It can be induced that the Ni content has little influence on the hardness and yield strength of alloys before aging treatment. It can be found that the hardness and yield strength of alloys after aging treatment vary significantly with the increase of Ni content. It can be induced that the ratio of Ni and Sn of alloy after aging treatment is responsible for the improvement of the hardness and yield strength.

**Figure 3:**
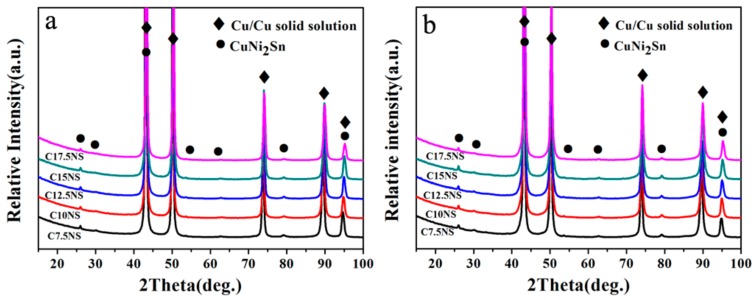
X-ray diffraction (XRD) patterns of the studied alloys with different Ni contents (**a**) before aging treatment; (**b**) after aging treatment.

The X-ray diffraction (XRD) patterns of the alloys before and after aging treatment are shown in Figure 3. As shown in Figure 3a,b, the phase constituents of the alloys before and after aging treatment are not obvious different and basically similar. The phase constituents of the alloys before and after aging treatment are mainly composed of Cu/Cu solid solution and CuNi_2_Sn. Results show that the phase compositions of the alloys before and after aging treatment have no distinct changes with the increase of Ni content. It can be deduced that the CuNi_2_Sn form during the cooling after sintering.

### 3.2. Microstructure and Morphology of the Alloys

**Figure 4:**
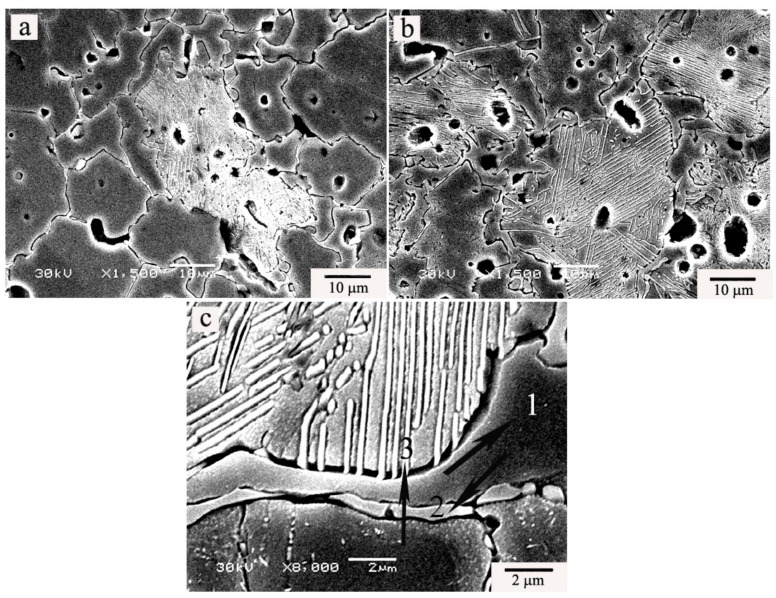
Typical scanning electron microscopy (SEM) secondary electron (SE) images of alloys: Cu-12.5Ni-5Sn alloy before (**a**) and after (**b**) aging treatment; and, (**c**) the SEM-SE image of Cu-12.5Ni-5Sn alloy after aging treatment.

Figure 4 shows the typical scanning electron microscopy (SEM) micrographs of Cu-12.5Ni-5Sn alloy before and after aging treatment. It can be observed from Figure 4 that precipitates form at the grain boundary and in the grain, and the variation of precipitates of the Cu-12.5Ni-5Sn alloy before and after aging treatment is distinct. Before aging treatment, small amounts of precipitated phases are found, as shown in Figure 4a. After aging treatment, large amounts of precipitated phases were observed, as shown in Figure 4b. This may reveal that the aging treatment can be contributed to the formation of precipitated phases and the increase of precipitates contributes to the improvement of mechanical properties. The precipitates at the grain boundary are mainly in the elongated shape and the precipitates in the grain are mainly the lamellar shape and nearly engulf the whole grain, as shown in Figure 4b,c. The results of EDS analysis near the locations, as pointed by arrows in Figure 4c, are listed in Table 2. The phase composition as pointed by arrow 1 is similar to the nominally chemical composition, but the precipitate at the grain boundary as pointed by arrow 2 is rich in both Ni and Sn, which the ratio of Ni to Sn is almost 1:1, and the precipitate in the grain, as pointed by arrow 3, is also rich in both Ni and Sn, which the percent of Ni and Sn can reach about 18 wt % and 15 wt %, respectively.

**Figure 5:**
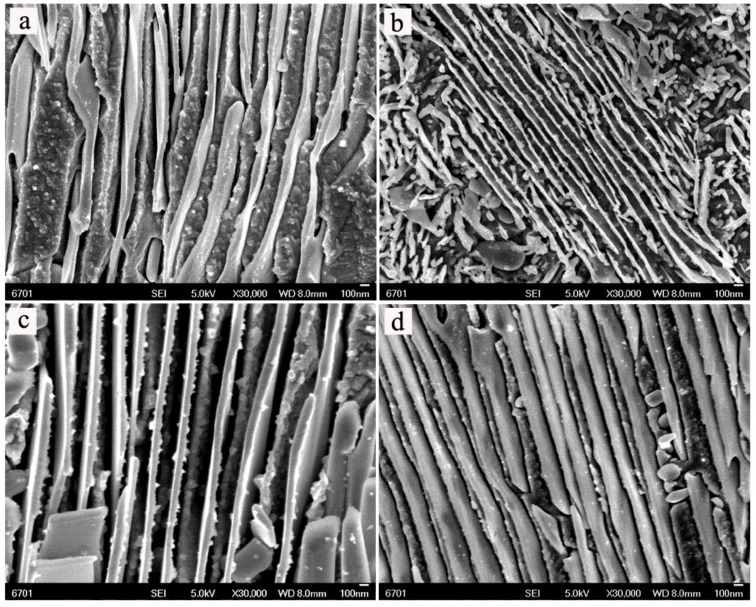
Typical field emission scanning electron microscope (FESEM) micrographs of the alloys before aging treatment for (**a**) Cu-12.5Ni-5Sn, and after aging treatment for (**b**) Cu-7.5Ni-5Sn, (**c**) Cu-12.5Ni-5Sn, and (**d**) Cu-17.5Ni-5Sn.

**Figure 6:**
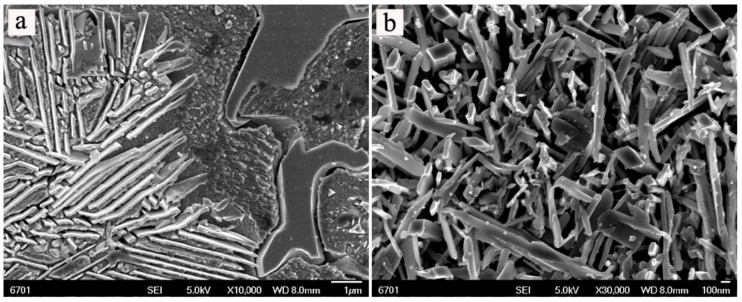
Typical FESEM micrographs of the Cu-12.5Ni-5Sn alloy after aging treatment: (**a**) the lamellar precipitates grow and consume matrix; (**b**) the morphology of needle-like precipitates.

In order to study the morphologies of the alloys, the high magnification micrographs were examined by field emission scanning electron microscope (FESEM). Figure 5 shows the typical FESEM images of the alloys with different Ni contents before and after aging treatment. From Figure 5, the formation of lamellar precipitates in the matrix generates the sandwich structures. From Figure 5b–d, the morphology of the alloy after aging treatment is slightly different with the increasing of Ni content. It can be found that the lamellar precipitates appear to coarsen and basically exhibit a trend of increase with increasing Ni content. From Figure 5a,c, the sandwich structure after aging treatment seem to be more inerratic when compared with those before aging treatment. The growth of lamellar precipitates during aging gradually consumes the matrix of Cu solid solution, as shown in Figure 6a. Figure 6b shows that the needle-like phase is found in the Cu-12.5Ni-5Sn alloy after aging treatment, which may contribute to enhancing the mechanical properties of the alloy, as discussed by Spooner [7]. It can be deduced that the sandwich structure may contribute to improve the mechanical properties of Cu-Ni-Sn alloys, but the excessive lamellae in the sandwich structure may be harmful to mechanical properties of the alloys.

Lefevre et al. [6] reported that strengthening of Cu-Ni-Sn alloy derived from spinodal decomposition. Rhu et al. [8] deemed that age-hardening came from DO_22_ precipitated phase. There is no agreement on the strengthening mechanism of Cu-Ni-Sn alloys. In the paper, it is found that the formation of lamellar precipitates may be responsible for the improvement of mechanical properties of the Cu-Ni-Sn alloys after aging treatment. As shown Figure 3 and Figure 4, the precipitates are found in alloys before and after aging treatment, but the precipitates in alloy before aging treatment are few. So, it can be induced that the alloys may generate lamellar precipitates during the cooling after sintering. The lamellar precipitates have not been able to grow because the time is short during the cooling. So, the lamellar precipitates in alloy before aging treatment have no significant influence on the mechanical properties. Lefevre et al. [6] deemed that the lamellar precipitates derive from the spinodal structure. So, it is deduced that the phase between the lamellar precipitates may be a spinodal structure. From Figure 4 and Figure 5, the lamellar precipitates and spinodal structures generate the sandwich structures. The amount of sandwich structures obviously increases after aging treatment. From Figure 2, Figure 4 and Figure 5, the lamellar precipitates in sandwich structure is more inerratic and denser with the increase of Ni content after aging treatment because the precipitated phases are rich both Ni and Sn. It can be found that the improvement of mechanical properties can attribute to the increase of lamellar precipitates, but it is not a positive correlation between mechanical properties and lamellar precipitates. The mechanical properties reach up to the maximum when the Ni content is 12.5%. It can be deduced that the amount of lamellar precipitates in a sandwich structure after aging treatment may be determined by the ratio of Ni and Sn, and the mechanical properties may be governed by the amount of lamellar precipitates, and keeping balance between the lamellar precipitates and spinodal structures in sandwich structure can be responsible for the improvement of mechanical properties of Cu-Ni-Sn alloys, which is in agreement with the report of Lefevre et al. [6] and Sadi et al. [3].

## 4. Conclusions

1. The hardness and yield strength of the Cu-Ni-5Sn alloys firstly increase and then decrease with the increase of Ni content, and reach up to a maximum when Ni content is 12.5 wt %.
2. The sandwich structures and needle-like phase are observed by FESEM, and the grain boundary and intragranular precipitates are rich in both Ni and Sn.
3. The formation of the inerratic and suitable lamellar precipitates in a sandwich structure and needle-like phase can be responsible for the good mechanical properties of the Cu-12.5Ni-5Sn alloy after aging treatment.

## Figures and Tables

**Table 1:** Nominally chemical composition, density of the alloys.

Sample	Nominally Chemical Composition (wt %)			Theoretical Density (g/cm^3^)	Measured Density (g/cm^3^)
	Cu	Ni	Sn		
C7.5NS	87.5	7.5	5	8.85	7.63
C10NS	85	10	5	8.85	7.79
C12.5NS	82.5	12.5	5	8.85	7.82
C15NS	80	15	5	8.85	7.90
C17.5NS	77.5	17.5	5	8.85	7.92

**Table 2:** The energy dispersive spectroscopy (EDS) analysis near the locations pointed by black arrows in Figure 4c (wt %).

Element	1	2	3
Cu	83.62 ± 0.86	44.54 ± 9.95	66.48 ± 4.41
Ni	10.52 ± 0.31	25.43 ± 4.15	18.13 ± 2.16
Sn	5.87 ± 1.14	25.84 ± 5.80	15.39 ± 2.33

## References

[1] Plewes J.T. (1975). High-strength Cu-Ni-Sn alloys by thermomechanical processing. Metall. Trans. A.

[2] Zhao J.C., Notis M.R. (1998). Microstructure and precipitation in a Cu-7.5Ni-5Sn alloy. Scr. Mater..

[3] Sadi F., Servant C. (2007). Phase transformations and phase diagram at equilibrium in the Cu-Ni-Sn system. J. Therm. Anal. Calorim..

[4] Hermann P.H., Morris D.G. (1994). Relationship between microstructure and mechanical properties of a spinodally decomposing Cu-15Ni-8Sn alloy prepared by spray deposition. Metall. Mater. Trans. A.

[5] Singh J.B., Cai W., Bellon P. (2007). Dry sliding of Cu–15 wt % Ni–8 wt % Sn bronze: Wear behaviour and microstructures. Wear.

[6] Lefevre B.G., D’Annessa A.T., Kalish D. (1978). Age hardening in Cu-15Ni-8Sn alloy. Metall. Trans. A.

[7] Spooner S., Lefevre B.G. (1980). The effect of prior deformation on spinodal age hardening in Cu-15 Ni-8 Sn alloy. Metall. Trans. A.

[8] Rhu J.C., Kim S.S., Jung Y.C., Han S.Z., Kim C.J. (1999). Tensile strength of thermomechanically processed Cu-9Ni-6Sn alloys. Metal. Mater. Trans. A.

[9] Kim S.S., Rhu J.C., Jung Y.C., Han S.Z., Kim C.J. (1999). Aging characteristics of thermomechanically processed Cu-9Ni-6Sn alloy. Scr. Mater..

[10] Diánez M.J., Donoso E., Sayagués M.J., Perejón A., Sánchez-Jiménez P.E., Pérez-Maqueda L.A., Criado J.M. (2016). The calorimetric analysis as a tool for studying the aging hardening mechanism of a Cu-10wt %Ni-5.5wt %Sn alloy. J. Alloys Compd..

[11] Zhao J.C., Notis M.R. (1998). Spinodal decomposition, ordering transformation, and discontinuous precipitation in a Cu–15Ni–8Sn alloy. Acta Mater..

[12] Peng G., Gan X., Jiang Y., Li Z., Zhou K. (2017). Effect of dynamic strain aging on the deformation behavior and microstructure of Cu-15Ni-8Sn alloy. J. Alloys Compd..

[13] Hui Z., Yizhu H., Xiaomin Y., Ye P. (2010). Microstructure and age characterization of Cu-15Ni-8Sn alloy coatings by laser cladding. Appl. Surf. Sci..

[14] Virtanen P., Tiainen T., Lepistö T. (1998). Precipitation at faceting grain boundaries of Cu–Ni–Sn alloys. Mater. Sci. Eng. A.

[15] Caris J., Varadarajan R., Stephens J.J., Lewandowski J.J. (2008). Microstructural effects on tension and fatigue behavior of Cu–15Ni–8Sn sheet. Mater. Sci. Eng. A.

[16] Alili B., Bradai D., Zieba P. (2008). On the discontinuous precipitation reaction and solute redistribution in a Cu-15%Ni-8%Sn alloy. Mater. Charact..

[17] Caris J., Li D., Stephens J.J., Lewandowski J.J. (2010). Microstructural effects on tension behavior of Cu–15Ni–8Sn sheet. Mater. Sci. Eng. A.

[18] Das A., Verma V., Basak C.B. (2016). Elucidating microstructure of spinodal copper alloy through annealing. Mater. Charact..

[19] Sahu P., Pradhan S.K., De M. (2004). X-ray diffraction studies of the decomposition and microstructural characterization of cold-worked powders of Cu-15Ni-Sn alloys by Rietveld analysis. J. Alloys Compd..

[20] Shankar K.V., Sellamuthu R. (2015). Determination of the Effect of Nickel Content on Hardness, Optimum Aging Temperature and Aging Time for Spinodal Bronze Alloys Cast in Metal Mould. Appl. Mech. Mater..

[21] Shankar K.V., Sellamuthu R. (2017). Determination on the effect of tin content on microstructure, hardness, optimum aging temperature and aging time for spinodal bronze alloys cast in metal mold. Int. J. Metalcast..

